# Correction: Platinum nanoparticles inhibit bacteria proliferation and rescue zebrafish from bacterial infection

**DOI:** 10.1039/d3ra90068a

**Published:** 2023-08-04

**Authors:** Khan Behlol Ayaz Ahmed, Thiagarajan Raman, Veerappan Anbazhagan

**Affiliations:** a Department of Chemistry, SASTRA University Thanjavur 613401 Tamil Nadu India anbazhagan@scbt.sastra.edu +91-04362-264120 +91-04362-264101-3689; b Department of Bioengineering, School of Chemical & Biotechnology, SASTRA University Thanjavur 613401 Tamil Nadu India raman@scbt.sastra.edu +91-04362-264120 +91-04362-264101-2359

## Abstract

Correction for ‘Platinum nanoparticles inhibit bacteria proliferation and rescue zebrafish from bacterial infection’ by Khan Behlol Ayaz Ahmed *et al.*, *RSC Adv.*, 2016, **6**, 44415–44424, https://doi.org/10.1039/C6RA03732A.

The authors regret that the image representing a double dose of PtNPs at 15 h in Fig. 3 was shown incorrectly in the original article. The correct version of Fig. 3 is shown below.

**Fig. 1:**
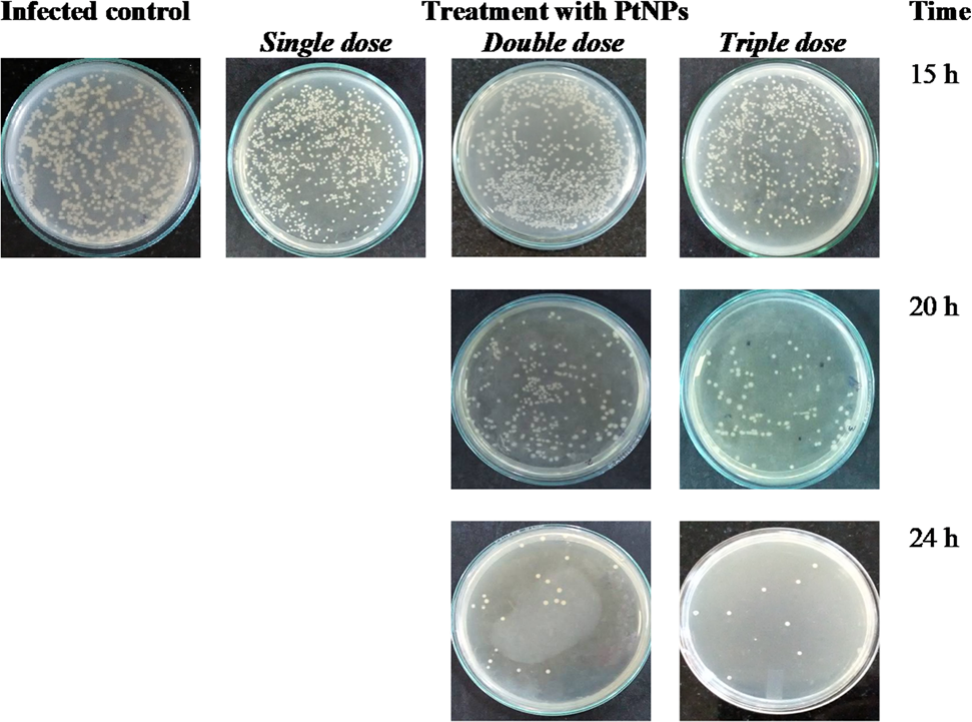
Multiple dose treatment. *E. coli*-infected fish were treated with three doses of PtNPs. Each dose consists of 10 μL of 0.1 mM PtNPs. Muscle tissue was dissected at the reported time point and homogenized and plated on the sterile LB agar plates. With the single-dose treatment, no fish are alive after 15 h, so we don’t show the plating.

The Royal Society of Chemistry apologises for these errors and any consequent inconvenience to authors and readers.

## Supplementary Material

